# Genomic Prediction Using LD-Based Haplotypes Inferred From High-Density Chip and Imputed Sequence Variants in Chinese Simmental Beef Cattle

**DOI:** 10.3389/fgene.2021.665382

**Published:** 2021-07-29

**Authors:** Hongwei Li, Bo Zhu, Ling Xu, Zezhao Wang, Lei Xu, Peinuo Zhou, Han Gao, Peng Guo, Yan Chen, Xue Gao, Lupei Zhang, Huijiang Gao, Wentao Cai, Lingyang Xu, Junya Li

**Affiliations:** ^1^Laboratory of Molecular Biology and Bovine Breeding, Institute of Animal Sciences, Chinese Academy of Agricultural Sciences, Beijing, China; ^2^National Centre of Beef Cattle Genetic Evaluation, Beijing, China; ^3^College of Computer and Information Engineering, Tianjin Agricultural University, Tianjin, China

**Keywords:** genomic prediction, prediction accuracy, LD, haplotype, Chinese Simmental beef cattle

## Abstract

A haplotype is defined as a combination of alleles at adjacent loci belonging to the same chromosome that can be transmitted as a unit. In this study, we used both the Illumina BovineHD chip (HD chip) and imputed whole-genome sequence (WGS) data to explore haploblocks and assess haplotype effects, and the haploblocks were defined based on the different LD thresholds. The accuracies of genomic prediction (GP) for dressing percentage (DP), meat percentage (MP), and rib eye roll weight (RERW) based on haplotype were investigated and compared for both data sets in Chinese Simmental beef cattle. The accuracies of GP using the entire imputed WGS data were lower than those using the HD chip data in all cases. For DP and MP, the accuracy of GP using haploblock approaches outperformed the individual single nucleotide polymorphism (SNP) approach (GBLUP_In_Block) at specific LD levels. Hotelling’s test confirmed that GP using LD-based haplotypes from WGS data can significantly increase the accuracies of GP for RERW, compared with the individual SNP approach (∼1.4 and 1.9% for G_H_BLUP and G_H_BLUP+GBLUP, respectively). We found that the accuracies using haploblock approach varied with different LD thresholds. The LD thresholds (*r*^2^ ≥ 0.5) were optimal for most scenarios. Our results suggested that LD-based haploblock approach can improve accuracy of genomic prediction for carcass traits using both HD chip and imputed WGS data under the optimal LD thresholds in Chinese Simmental beef cattle.

## Introduction

Genomic prediction (GP) has been widely used in the past decades (Meuwissen et al., 2001). Many approaches, including GBLUP (VanRaden, 2008), Bayes alphabet (Habier et al., 2011; Gianola, 2013), and machine learning (Li et al., 2018; Yin et al., 2020), have been proposed to improve prediction accuracy. Most of these approaches were developed based on single nucleotide polymorphisms (SNPs). Genomic prediction using haplotypes instead of SNPs can be more accurate (Zondervan and Cardon, 2004). A haplotype is defined as a combination of alleles at adjacent loci belonging to the same chromosome that are transmitted as a unit (Vormfelde and Brockmöller, 2007; Won et al., 2020) and a haplotype may contain the combined effects of causal variants with high linkage disequilibrium (LD) (Balding, 2006; Garnier et al., 2013), thus this approach can effectively identify the loci with small effects, which may not be captured by a single marker (Feitosa et al., 2020).

Many previous studies have shown that genomic selection using haplotypes is more reliable than that using individual SNPs for both simulated and real data, even when the marker density is low (Calus et al., 2008; De Roos et al., 2008). Cuyabano et al. (2014) compared the genomic predictions between the haplotype-based (constructed based on LD and using HD chip data) and the SNP-based approach for milk production and health traits in dairy cattle, suggesting the high prediction ability using the haplotype-based approach. Moreover, Hess et al. (2017) found that fitting covariates for haplotype alleles instead of SNPs can increase the prediction accuracy up to 5.5% (Hess et al., 2017). Recently, Xu et al. (2020) reported that the haplotype-based model using HD chip data can improve the accuracy by 5.4–9.8%, compared with the SNP-based approach for carcass and live weight traits.

Haploblocks can be constructed through multiple strategies including the fixed block length based on centimorgans (cM) (Boichard et al., 2012), base pairs (bp) (Sun, 2016), or a constant number of SNPs per block (Hayes et al., 2007; Calus et al., 2009; Villumsen et al., 2009) and not fixed length approach based on the LD pattern (Cuyabano et al., 2015). Many improved methods have been proposed to account for recombination hotspots and coldspots across the genome (Calus et al., 2008; Sandor et al., 2012; Weng et al., 2014; Cuyabano et al., 2015). Haploblock construction based on the LD is expected to achieve a high prediction accuracy by selecting the effective SNPs and reducing the amount of predictor variables in the model (Cuyabano et al., 2015).

The WGS data can provide more potential causative polymorphisms, thus imputation from low density marker panels to WGS for datasets with a large number of individuals may be an effective approach to increase the accuracy of GP (Marchini et al., 2007; Browning and Browning, 2009; Howie et al., 2009; Li et al., 2010). A recent study suggested that genomic prediction within-population using simulated WGS data can increase (∼31%) the accuracy of prediction for traits with low and moderate heritability (Iheshiulor et al., 2016). Similarly, Druet et al. (2014) suggested that the prediction accuracy using simulated sequence data can be improved (∼30%) when including causal mutations with low minor allele frequencies. A previous study suggested that the haploblock approach may play an important role in the genomic prediction involving genome sequences (Cuyabano et al., 2014). The haploblocks containing additional markers are likely to be generated from WGS, which may reduce the number of variables compared with SNP and keep all SNP information. The haplotype approach based on WGS is likely to improve the accuracy of GP. However, the evaluations of prediction accuracies on the economically important traits using this strategy are still yet to be explored in cattle.

The objectives of current study were to (1) evaluate the predictive performance of carcass traits using HD chip and WGS data in Chinese Simmental beef cattle; (2) compare the differences of predictive accuracies between haplotype-based prediction model (G_H_BLUP), SNP-based prediction model (GBLUP), and the combination of haplotype and SNP prediction model (G_H_BLUP+GBLUP); and (3) investigate the LD-based haplotypes with different thresholds on the prediction accuracies.

## Materials and Methods

### Ethics Statement

All animals used in the study were treated following the guidelines established by the Council of China Animal Welfare. The procedure for collecting cattle blood samples and phenotypes was carried out in strict accordance with the protocol approved by the Science Research Department of the Institute of Animal Sciences, Chinese Academy of Agricultural Sciences (CAAS) (Beijing, China).

### Data

Data available comprised a total of 1,233 Simmental cattle born between 2008 and 2015 from Ulgai, Xilingol League, and Inner Mongolia, China. After weaning, cattle were moved to Jinweifuren Co., Ltd. (Beijing, China) for fattening under the same feeding and management conditions. A more detailed description of the management processes was reported in previous studies (Zhu et al., 2016, 2017). All individuals were slaughtered at an average age of 20 ± 2.2 months. Carcass and meat quality traits were measured in accordance with the guidelines proposed by the Institute of Meat Purchase Specifications established by the Agricultural Marketing Service of the USDA. From these traits, dressing percentage (DP), meat percentage (MP), and rib eye roll weight (RERW) were analyzed.

### Genotyping and Imputation

The DNA samples from blood were genotyped with Illumina BovineHD BeadChip. Before statistical analysis, the original SNP dataset was filtered using PLINK (v1.07) (Purcell et al., 2007; Chang et al., 2015). Individuals and autosomal SNPs were filtered by the following criteria: SNP call rate (<0.90), minor allele frequency (MAF < 0.01), Hardy–Weinberg equilibrium (*p* < 10^–6^), and individual call rate (<0.90). Missing genotypes were imputed using BEAGLE (v4.1) (Browning and Browning, 2016). Consequently, 1,233 individuals and 671,164 SNPs remained.

Forty-four unrelated individuals (according to the pedigree and PI-HAT value estimated using PLINK v1.07) were selected as the reference population for imputation. The whole genome sequencing of these individuals was performed using Illumina Hiseq2500 instruments (Illumina Inc., San Diego, CA, United States). All processes were performed according to the standard manufacturer’s protocols.

The SNPs from the HD chip were imputed to the sequencing level using BEAGLE (v4.1) (Browning and Browning, 2016). The imputed WGS was filtered by removing SNPs with a MAF less than 0.05. After quality control, a total of 6,776,719 SNPs remained. The imputation accuracy was assessed by the allelic R-squared measure (AR^2^), which is an estimate of the squared correlation between the most probable and the true reference dose. The average imputation accuracy was 0.83 when the MAF was larger than 0.05.

### Heritability and Variance Component Estimation

Phenotypes were adjusted for the fixed effects, including sex, year, and the covariates of body weight upon entering the fattening farm, and the number of fattening days. Subsequently, the adjusted phenotypes were used for further analyses. Variance components were estimated using the following univariate animal model in ASREML (v4.1).

(1) $$\text{y}=1_{n}\,\mu +\text{Za}+\text{e}$$

where **y** is the vector of the adjusted phenotypes, **1_n_** is an n × 1 vector with entries equal to 1; μ is the overall mean; $\text{a}∼N\,\left(0,\sigma_{a}^{2}\,\text{G}\right)$ is a vector of random additive genetic effect, where **G** is the additive genomic relationship matrix constructed using all SNPs and $\sigma_{e}^{2}$ is the additive genetic variance, **Z** is incidence matrix linking **a** to **y**; and $\text{e}∼N\,\left(0,\sigma_{e}^{2}\,\text{I}\right)$ is a vector of random residuals, where **I** is the identity matrix and $\sigma_{e}^{2}$ is the residual variance. The heritability estimates were calculated as ***h***=2σa2/(σa2+σe2).

### Haplotype Construction

The LD-based haploblocks were generated separately for each chromosome. A group of SNPs was defined as a haploblock if the LD between every two SNPs in the group was greater than or equal to the threshold value (*r*^2^). For two bi-allelic loci (*A*_1_/*A*_2_ *and B*_1_/*B*_2_), *r*^2^ was calculated as,

(2) $${\text{r}}^{2}=\frac{{\text{D}}^{2}}{\left({\text{p}}_{A_{1}}\,{\text{p}}_{A_{2}}\,{\text{p}}_{B_{1}}\,{\text{p}}_{B_{2}}\right)}$$

where ***D** = **p**_**A**_1_**B**_1__**p**_**A**_2_**B**_2__* − ***p**_**A**_1_**B**_2__**p**_**A**_2_**B**_1__*.

Seven different LD levels (*r*^2^) (0.2, 0.3, 0.4, 0.5, 0.6, 0.7, and 0.8) were set as the thresholds in this study.

Haplotype effects were modeled using numerical dosage coding strategies (Calus et al., 2008; Cuyabano et al., 2014, 2015; Meuwissen et al., 2014; Da, 2015). Numerical dosage coding of a haploblock is formed by two consecutive SNPs (Table 1). In the numerical dosage model, artificial SNPs were created for each haploblock, and these “SNPs” were coded as the number of copies.

**TABLE 1:** Numerical dosage coding of a haploblock formed by two consecutive single nucleotide polymorphisms (SNPs).

Haplotype allele 1	Haplotype allele 2	Numerical coding of haploblock			
		AB	Ab	aB	ab
AB	AB	2	0	0	0
AB	Ab	1	1	0	0
AB	aB	1	0	1	0
AB	ab	1	0	0	1
Ab	Ab	0	2	0	0
Ab	aB	0	1	1	0
Ab	ab	0	1	0	1
aB	aB	0	0	2	0
aB	ab	0	0	1	1
ab	ab	0	0	0	2

*AB, Ab, aB, and ab are four haplotype alleles of the same haploblock.*

### Genomic Prediction Models

The genomic best linear unbiased prediction (GBLUP) model including the haplotype/SNP effect was used for DP, MP, and RERW as described in Eq. (1). Three approaches based on (a) the SNPs, (b) the haploblock only, and (c) the haploblock and the non-blocked SNPs were considered for predictions. Seven different *r*^2^ thresholds were used for haploblock construction.

We performed genomic prediction using GBLUP for all SNP markers, and the genomic relationship matrix was calculated as $G=\frac{\left(M-P\right)\,{\left(M-P\right)}^{\prime }}{2\,\sum_{i=1}^{m}p_{i}\,\left(1-p_{i}\right)}$, where **M** denotes the (0, 1, 2)-encoded genotype matrix, *p_i_* is the MAF of marker *i*, *m* is the number of markers, and **P** is a matrix with columns equal to *2p_i_*.

Genomic prediction using GBLUP for the SNP markers inside of the block in HD chip and WGS data were defined as GBLUP_770K_In_Block and GBLUP_WGS_In_Block, respectively.

The haplotype-based genomic best linear unbiased prediction (G_H_BLUP) was performed for all markers. The haplotype-based genomic relationship matrix in G_H_BLUP was constructed as the product of the haplotype allele matrix (**M_H_**) and expressed as GH=MH⁢MH′QH, where **M_H_** is the pseudo-markers matrix with entries 0, 1, and 2 representing the number of copies of each haplotype allele in a haploblock, and Q_H_ is the total number of haplotype alleles of whole genome. In the G_H_BLUP+GBLUP model:

(3) $$y=1_{n}\,\mu +\text{Za}+{\text{Z}}_{u}\,{\text{a}}_{u}+\text{e}$$

which included the haploblock effects and the SNP effects estimated from outside the haploblocks (non-blocked SNPs). $\text{a}:N\,\left(0,\sigma_{a}^{2}\,{\text{G}}_{H}\right)$ is a vector of random additive genetic effect, where ***G_H_*** is the additive genetic relationship matrix constructed using haploblock and $\sigma_{a}^{2}$ is the additive genetic variance based on the haploblock, ***Z*** is incidence matrix associating ***a***; ${\text{a}}_{u}:N\,\left(0,\sigma_{a_{u}}^{2}\,\text{G}\right)$ is a vector of random additive genetic effect, where ***G*** is the additive genetic relationship matrix constructed using non-blocked SNPs and $\sigma_{a_{u}}^{2}$ is the additive genetic variance based on the haploblock, ***Z_u_*** is incidence matrix associating ***a_u_***; ***a*** is composed of haploblock effects and ***a_u_*** is composed of SNP effects estimated from outside the haploblocks. Also, they are considered as uncorrelated effects.

### Assessment of Prediction Accuracy

The accuracy of genomic prediction was assessed using fivefold cross-validation (CV). The CV procedure was applied by assigning animals randomly into five separate subsets. This procedure was randomly repeated 10 times.

The regression coefficient of the adjusted phenotype on GEBVs for individuals in the validation set was obtained to measure the degree of inflation/deflation of prediction, which was defined as follows:

(4) $$\text{b}=\frac{\text{Cov}\,\left(\mathrm{gebv},{\text{y}}^{{}^{*}}\right)}{\text{var}\,\left(\mathrm{gebv}\right)}$$

The average Pearson correlation coefficient between the adjusted phenotypic values and genomic estimated breeding values (GEBVs) in the validation set divided by square root of heritability was used as a measurement of prediction accuracy. The prediction accuracy was calculated as (Bolormaa et al., 2013):

(5) $$\mathrm{Prediction}\,\mathrm{accuracy}=\frac{\text{cor}\,\left({\text{y}}^{*},\mathrm{gebv}\right)}{\sqrt{{\text{h}}^{2}}}$$

where *y*^∗^ is adjusted phenotypic values, *gebv* is the genomic estimated breeding values (GEBVs), and *h*^2^ is the heritability.

To compare the differences of the accuracies of GP using three approaches (GBLUP, G_H_BLUP, and G_H_BLUP+GBLUP) and marker densities (HD chip and WGS), we used Hotelling’s (1940) *t* statistic (Hotelling, 1940) to test the significance of the differences.

The test statistic ***t*** is given by,

(6) $$t=\frac{\left({\text{r}}_{\mathrm{jk}}-{\text{r}}_{\mathrm{jh}}\right)\,\sqrt{\left(\text{n}-3\right)\,\left(1+{\text{r}}_{\mathrm{kh}}\right)}}{\sqrt{2\,\left|R\right|}}$$

with *df* = *n* − 3, where,

(7) $$\left|R\right|=1+2\,r_{j\,k}\,r_{j\,h}\,r_{k\,h}-r_{j\,k}^{2}-r_{j\,h}^{2}-r_{k\,h}^{2}$$

where **r** is the observed correlation and *n* is the number of observations. For instance, while comparing the differences of accuracy between the GBLUP and G_H_BLUP, the ***r_jk_*** is the cor(*y*^∗^,*gebv_GBLUP_*), the ***r_jh_*** is the *cor*(*y*^∗^,*gebv*_*G_H_BLUP*_), and the ***r_kh_*** is the *cor*(*gebv_GBLUP_*,*gebv_GHBLUP_*). If ***P***(*T* ≥ *t*) ≤ α(α =  0.05), then the hypothesis (*H*_0_:*r_jk_* = *r_jh_*) is rejected. Hence, we can conclude whether correlations were significantly different.

## Results

### Heritability Estimation and Haploblock Construction

Based on the HD chip data, the estimated heritabilities of DP, MP, and RERW using univariate animal model were 0.27, 0.17, and 0.23, respectively, and the statistical description is shown in Table 2. Notably, under threshold *r*^2^ > 0.2, we observed 68,775 (362,710 SNPs) and 634,662 (3,536,404 SNPs) blocks from the HD chip and WGS data, while the number of SNPs out of blocks were 298,454 and 3,240,315 and haplotype allele counts were 840,676 and 3,370,157. Details about the total number of haplotype alleles (variables), haploblocks, and non-blocked SNPs with different *r*^2^ are presented in Table 3. The number of haplotype alleles and haploblocks decreases with increasing *r*^2^. The average number of SNPs per haploblock ranged from 3.3 to 5.3 for the HD chip data and from 4.5 to 5.6 for the WGS data. According to our results, we found that the method based on haploblock reduced the number of variables (haplotype alleles) for the WGS data. However, as for the HD chip data, the haploblock approach increased the number of variables compared with the SNP approach. This result mainly depends on the data type used for haploblock construction (HD or WGS).

**TABLE 2:** Statistical description and heritability estimation of three traits in Chinese Simmental beef cattle.

Trait^1^	Number of phenotypes	Mean ± SD	Maximum	Minimum	h^2^ ± SE
DP	1,221	0.535 ± 0.029	0.690	0.410	0.27 ± 0.07
MP	1,226	0.456 ± 0.031	0.616	0.325	0.17 ± 0.06
RERW	1,228	10.67 ± 2.20	18.32	5.03	0.23 ± 0.06

*^1^Trait: DP, dressing percentage; MP, meat percentage; RERW, rib eye roll weight.*

**TABLE 3:** Total number of haplotype alleles, haploblocks, and the non_blocked SNPs from the 770K array and sequence data.

Data	*r* ^2^1^^	Haplotype alleles	Haploblocks (Blocked_SNPs)	Number of SNPs per haploblock	Non_blocked SNPs
770K	0.2	840,676	68,775 (362,710)	5.3	298,454
	0.3	599,270	66,027 (309,126)	4.7	352,038
	0.4	462,287	62,150 (268,055)	4.3	393,109
	0.5	371,074	58,009 (234,305)	4.0	426,859
	0.6	303,774	53,876 (204,725)	3.8	456,439
	0.7	249,195	49,320 (176,622)	3.6	484,542
	0.8	199,702	43,892 (147,008)	3.3	514,156
WGS	0.2	3,370,157	634,662 (3,536,404)	5.6	3,240,315
	0.3	2,701,350	601,761 (3,142,601)	5.2	3,634,118
	0.4	2,323,965	571,908 (2,877,612)	5.0	3,899,107
	0.5	2,067,572	545,069 (2,676,430)	4.9	4,100,289
	0.6	1,866,317	522,099 (2,502,764)	4.8	4,273,955
	0.7	1,684,138	500,294 (2,329,147)	4.7	4,447,572
	0.8	1,493,399	477,242 (2,123,952)	4.5	4,652,767

*^1^Seven different LD thresholds set from r^2^ ≥ 0.2 to r^2^ ≥ 0.8.*

We also evaluated the LD decay between 0 and 100 kb for BTA1 in the HD chip and WGS data, respectively. The average *r*^2^ was calculated for each 1-kb window size. LD decay suggested that the HD chip data had a faster LD decay than WGS data (Supplementary Figure 1), thus prediction accuracies using the HD chip data among different LD thresholds displayed obvious changes compared with the WGS data. We observed *r*^2^ decreased from 0.8 to 0.2 as the marker distances of the HD chip (from 0 to 35 kb) and WGS data (from 0 to 25 kb) increased. However, no obvious difference was found when *r*^2^ < 0.2. Therefore, we chose the LD thresholds (*r*^2^ ≥ 0.2 to *r*^2^ ≥ 0.8) to construct the haploblocks in our study.

### Genomic Prediction Accuracy

#### Comparison of Accuracies of GP Based on Three Different Approaches

**FIGURE 1:**
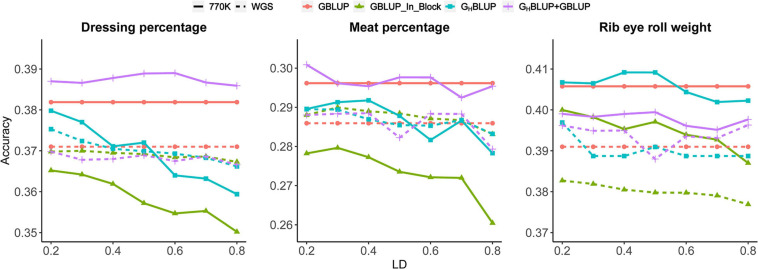
Prediction accuracies of different *r*^2^ thresholds for three traits based on the 770K data and WGS data.

Three different approaches, including (a) GBLUP, (b) G_H_BLUP, and (c) G_H_BLUP+GBLUP, were considered for the comparisons. As shown in Figure 1, G_H_BLUP+GBLUP had better performance for DP and MP than G_H_BLUP. We also found G_H_BLUP+GBLUP_770K yielded ∼1.8% higher accuracy than G_H_BLUP_770K for DP on average. However, as for the RERW, G_H_BLUP_770K had a slight higher accuracy than G_H_BLUP+GBLUP_770K, and the G_H_BLUP+GBLUP_WGS had better performance than GBLUP_WGS_In_Block.

To evaluate whether the observed differences were statistically significant, we compared the correlation of the prediction accuracies using Hotelling’s test. In the current study, we found no significant differences between the G_H_BLUP_770K and GBLUP_770K_In_Block for all scenarios (Table 4). However, G_H_BLUP_WGS using the WGS data had a significant improvement for RERW compared with GBLUP_WGS_In_Block (Table 5).

**TABLE 4:** *P*-values of Hotelling’s *t*-test comparing the prediction accuracy obtained with the individual SNP and haploblock approaches using 770K data.

*r* ^2^1^^	G_H_BLUP_770K			G_H_BLUP+GBLUP_770K		
	DP	MP	RERW	DP	MP	RERW
0.2	0.102	0.315	0.462	0.097	0.211	0.931
0.3	0.147	0.295	0.352	0.089	0.342	0.994
0.4	0.299	0.198	0.113	0.052	0.292	0.719
0.5	0.101	0.213	0.161	0.019	0.167	0.811
0.6	0.293	0.400	0.221	0.013	0.150	0.864
0.7	0.367	0.188	0.274	0.026	0.240	0.837
0.8	0.288	0.102	0.065	0.015	0.059	0.391

*^1^Seven different LD thresholds set from r^2^ ≥ 0.2 to r^2^ ≥ 0.8.*

**TABLE 5:** *P*-values of Hotelling’s *t*-test comparing the prediction accuracy obtained with the individual SNP and haploblock approaches using WGS data.

*r* ^2^1^^	G_H_BLUP_WGS			G_H_BLUP+GBLUP_WGS		
	DP	MP	RERW	DP	MP	RERW
0.2	0.311	0.835	0.012	0.999	0.998	0.040
0.3	0.643	0.938	0.218	0.717	0.837	0.047
0.4	0.852	0.769	0.142	0.829	0.946	0.588
0.5	0.908	0.670	0.047	0.939	0.357	0.161
0.6	0.869	0.796	0.111	0.902	0.875	0.095
0.7	0.956	0.997	0.088	0.997	0.833	0.074
0.8	0.833	0.992	0.036	0.936	0.628	0.025

*^1^Seven different LD thresholds set from r^2^ ≥ 0.2 to r^2^ ≥ 0.8.*

**FIGURE 2:**
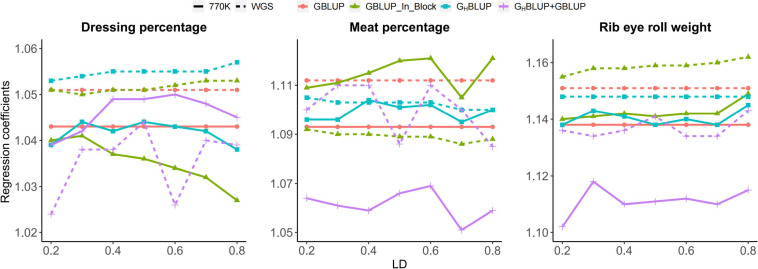
Regression coefficients of pre-adjusted phenotypes on GEBVs for three traits in Chinese Simmental beef cattle.

Accordingly, the slopes of the regression of the adjusted phenotype on GEBVs based on three approaches were presented in Figure 2. Our result showed that the regression coefficients of the HD chip were closer to 1 than those of the WGS data for most scenarios. The G_H_BLUP was near 1 for RERW when *r*^2^ ≥ 0.5. However, regression coefficients based on WGS data were almost stable for different LD levels.

#### Comparison of Accuracies of GP Based on Different Marker Densities

We found that genomic predictions using the HD chip were superior to the WGS data for all three traits (Figure 1); the accuracy of GBLUP_770K was 0.011, 0.01, and 0.015 higher than that of GBLUP_WGS for DP, MP, and RERW, respectively. However, no significant difference was found between the accuracies based on the two different densities according to Hotelling’s test (Table 6). Moreover, significant differences between the two marker densities were observed for RERW using both GBLUP and G_H_BLUP when SNPs within the blocks (divided by different LD thresholds) were selected. As for the G_H_BLUP+GBLUP, no significant differences were found between HD chip and WGS. It should be noted that the accuracies of the three traits decreased obviously for GBLUP_770K_In_Block compared with the GBLUP_770K (Figure 1). However, no obvious change was found between GBLUP_WGS and GBLUP_WGS_In_Block.

**TABLE 6:** *P*-values of Hotelling’s *t*-test comparing the prediction accuracy obtained with the 770K data and the WGS data.

*r* ^2^1^^	GBLUP			G_H_BLUP			G_H_BLUP+GBLUP		
	DP	MP	RERW	DP	MP	RERW	DP	MP	RERW
–	0.881	0.915	0.852	–	–	–	–	–	–
0.2	0.359	0.189	0.002	0.405	0.992	0.079	0.244	0.515	0.797
0.3	0.287	0.199	0.007	0.575	0.850	0.034	0.163	0.668	0.729
0.4	0.193	0.171	0.022	0.929	0.600	0.007	0.129	0.689	0.687
0.5	0.053	0.104	0.012	0.726	0.772	0.002	0.116	0.359	0.298
0.6	0.036	0.116	0.048	0.423	0.679	0.016	0.085	0.577	0.815
0.7	0.049	0.135	0.065	0.429	0.990	0.034	0.139	0.798	0.839
0.8	0.017	0.031	0.196	0.309	0.587	0.035	0.109	0.325	0.892

*^1^Seven different LD thresholds set from r^2^ ≥ 0.2 to r^2^ ≥ 0.8.*

#### Comparison of Accuracies of GP Among Different LD Levels

To investigate the influence of LD levels (*r*^2^) on the prediction accuracy, we constructed haploblocks (from *r*^2^ ≥ 0.2 to *r*^2^ ≥ 0.8) using seven different levels. In our study, we found that the haploblock approach (including G_H_BLUP and G_H_BLUP+GBLUP) was better than the individual SNP approach (GBLUP_In_Block) at specific LD thresholds (*r*^2^) (Figure 1). The accuracy of G_H_BLUP_770K showed the highest accuracy for RERW when *r*^2^ ≥ 0.5, and the G_H_BLUP_WGS outperformed GBLUP_WGS_In_Block. Under the strict LD threshold (*r*^2^), the G_H_BLUP+GBLUP_770K showed significant improvement compared with GBLUP_770K_In_Block for DP (Table 4).

### Computation Time

In our study, the average computation time of GBLUP, G_H_BLUP, and G_H_BLUP+GBLUP were 4.42, 5.41, and 41.5 min using fivefold cross-validation respectively (repeated 10 times). The time for constructing haplotype-based genomic relationship matrix (**G_H_**) were 8.22 and 203.97 h on average for the HD chip and WGS data, and 0.898 and 9.12 h for the SNP-based genomic relationship matrix (**G)**.

## Discussion

### Predictive Performance of Different Marker Density

In this study, we compared the accuracies of genomic prediction using both the HD chip and WGS data. A previous study suggested that prediction of breeding value was expected to be more accurate using the WGS data compared with the high-density chip because the causal mutations are assumed to be included in the WGS data (Druet et al., 2014). Genomic predictions based on sequence data can increase accuracy compared with predictions based on ∼30K SNP chips in simulation data (Meuwissen and Goddard, 2010; Clark et al., 2011; Druet et al., 2014; MacLeod et al., 2014). In contrast, for real data, a recent study found that no increases for prediction accuracy was observed using the imputed sequence data in Holstein Friesian cattle (Van Binsbergen et al., 2015). Our results presented the HD chip data had better performance than the WGS data using GBLUP approach. These findings can be explained by several factors including imputation accuracy, LD, MAF, genotyping errors, and population size (Iwata and Jannink, 2010; Zhang and Druet, 2010; Hayes et al., 2012; Ali et al., 2020). For instance, small reference population size and high imputation error rate from low-frequency SNPs may cause the decrease of accuracy for GP in WGS data (Heidaritabar et al., 2016). In addition, the strong LD between multiple true causal SNPs and potential QTLs segregating in long haplotypes in WGS data may make it difficult to pinpoint the truly causal SNP (Van Binsbergen et al., 2015).

### Comparison of Methods of Genomic Prediction Based on SNP Chip

For the HD chip data, our results showed that G_H_BLUP+GBLUP had the highest accuracy and G_H_BLUP was better than GBLUP at different LD levels for DP and MP (Figure 1). In contrast, G_H_BLUP showed the highest accuracy for RERW, which can be explained by the different genetic architectures of three traits. Moreover, DP and MP can be regarded as the compound traits, compared with RERW, which were determined by many genes with small effects. G_H_BLUP+GBLUP for these two traits can include the non-blocked SNPs in the model, which should be more effective to increase the prediction accuracy. However, G_H_BLUP+GBLUP approach may produce large prediction error variance and decrease the accuracy of GP for RERW due to the overestimation of the effects.

In addition, the G_H_BLUP+GBLUP approach may reflect the real genetic architectures of these traits. For instance, a gene region contains many consecutive loci, which can be effectively modeled by the G_H_BLUP approach. As for the gene regulatory region, the promoters or enhancers may be influenced by a single mutation, and this feature can be effectively integrated by the GBLUP approach.

Our findings were consistent with previous reports (Cuyabano et al., 2014; Teissier et al., 2020; Xu et al., 2020); they found that haplotype approach based on average LD threshold (*r*^2^ ≥ 0.45) can increase the prediction accuracies for milk production traits (up to 3.1%) compared with the individual SNP approach. Also, the accuracies of G_H_BLUP+GBLUP and G_H_BLUP can be influenced by the genetic architectures of different traits (Cuyabano et al., 2014).

The advantage of haplotype approach can be explained by the fact that SNPs are commonly bi-allelic, and SNP mutations in different loci tended to cause major changes in the haplotype frequencies (Curtis et al., 2001). Moreover, a QTL may be in complete LD with a multi-marker haplotype even if it is not in complete LD with any individual bi-allelic SNP marker (Cuyabano et al., 2014).

In our study, we found that the G_H_BLUP+GBLUP_770K showed the highest accuracy for DP and MP. The LD level was set to *r*^2^ ≥ 0.2 in the haplotype prediction and several SNPs showing weak LD (*r*^2^ from 0 to 0.2) with potential QTLs were not included in the model; therefore, adding the non-blocked SNPs may increase the prediction accuracy without loss of information. Also, we found that the regression coefficients using haplotype approach including G_H_BLUP+GBLUP and G_H_BLUP is close to 1 for all three traits, compared with SNP approach (Figure 2), which were consistent with the prediction accuracy using the average Pearson correlation coefficient between the adjusted phenotypic values and genomic estimated breeding values.

### Comparison of Methods of Genomic Prediction Based on WGS

As for the WGS data, our results suggested the haploblock approach based on LD can increase the accuracies of GP while reducing the number of variables. For RERW, G_H_BLUP_WGS and G_H_BLUP+GBLUP_WGS showed better performance than GBLUP_WGS_In_Block; however, no significant difference was found for DP and MP. The WGS data incorporating genotypes at causal variants into haplotypes allow effective estimation of haplotype effects. For DP and MP, we did not observe significant difference using both G_H_BLUP_WGS and G_H_BLUP+GBLUP_WGS. This result may be explained by the fact that the WGS data with high SNP density can produce the identified haplotype alleles (including some rare haplotype alleles); however, due to a large number of rare haplotype alleles with small effects or no effect were included in sequencing data (Gianola, 2013), the haplotype approach with them may not effectively improve prediction accuracy for DP and MP.

### Predictive Performance of Different LD Levels

In this study, we found that the prediction accuracies using haplotype approach varied among different LD thresholds for three traits, especially for the HD chip data. One possible reason is that the size of haploblocks varies among different LD thresholds and the QTL effects can be accurately estimated at specific LD levels because the effective haploblocks were included. The HD chip data may cause the loss of effective haploblock effects for genomic prediction compared with WGS data. However, no obvious difference among different LD thresholds using G_H_BLUP+GBLUP for three traits was observed. This result can be explained by the fact that G_H_BLUP+GBLUP approach contains both the haploblock effects and SNP effects which were estimated from outside haploblocks.

Similar as previous studies (Cuyabano et al., 2014, 2015; Feitosa et al., 2020), our study also revealed that the optimum LD threshold should be considered in the haplotype approach. For DP, the optimum LD threshold was *r*^2^ ≥ 0.2 (Figure 1). For MP and RERW, the optimum LD threshold appeared at *r*^2^ ≥ 0.5. Cuyabano et al., reported that haploblocks built based on *D*′ ≥ 0.45 can produce an optimal set of variables for milk protein, fertility, and mastitis traits. Our results indicated that the optimal thresholds of different traits are different. Therefore, it is hard to determine the optimal haploblock length for all scenarios. For instance, Villumsen et al. (2009) evaluated the optimal haploblock length for the simulated traits with heritabilities ranging from 0.02 to 0.30, they found that haploblocks of 1 cM (0.8 Mb) can produce the highest accuracies across all traits in New Zealand dairy cattle. Previous studies found that the optimal haploblock length ranged from 0.4 to 0.8 Mb per haploblock (Villumsen and Janss, 2009; Villumsen et al., 2009). Hess et al. (2017) found the highest prediction accuracy using short haploblock (250 kb) in the admixed dairy cattle population. Our study suggested the setting of optimal LD threshold depends on the LD between SNPs and QTLs and the population structure. Thus, the optimal LD threshold was required to be evaluated for each dataset independently.

It should be noted that haplotype approach based on LD had less improvement on the prediction accuracies compared with the fixed block length approach, which was in agreement with a previous study (Cuyabano et al., 2014). Xu et al. (2020) constructed the haploblock using the constant number of SNPs, and their findings suggested that the extension from the SNP-based model to haplotype-based model can improve the accuracy by 5.4–9.8%. Moreover, Hess et al. (2017) reported that fitting covariates for fixed-length haplotype alleles can increase the accuracy of GP up to 5.5% compared with SNPs.

In our study, we found that LD-based haplotype approach cannot increase the accuracy to 5%, which was consistent with a previous report (Cuyabano et al., 2014). They performed genomic prediction for three important traits (milk protein, fertility, and mastitis) using LD-based haplotypes in the Nordic Holstein population, and their finding suggested Bayesian model can produce the highest accuracy for the milk protein trait. This difference can be explained by the fact that all SNP information was included in the fixed block length approach, while only a small set of SNPs was included in the LD-based haplotype approach. In our study, we found that computation times were much longer for G_H_BLUP+GBLUP than GBLUP and G_H_BLUP, while no obvious difference was found between the GBLUP and G_H_BLUP approach. Two genomic relationship matrixes (**G_H_** and **G**) were estimated in the G_H_BLUP+GBLUP model, thus the long time was required for this approach. In addition, our results suggested that the haplotype approach for WGS data requires more time to construct the genomic relationship matrixes than the SNP-based approach. It should be noted that the haplotype-based genomic relationship matrix need to be recoded using numerical dosage coding strategies for each haploblock (Calus et al., 2008).

## Conclusion

Our study suggested that haploblock approach using both HD chip and WGS data can improve the prediction accuracy compared with the individual SNP approach. The prediction accuracies of haploblock approach varied in different LD thresholds. Therefore, it is important to determine the optimal *r*^2^ threshold when constructing haploblocks for genomic prediction. The advent of whole-genome sequencing has made it possible to contemplate linking the diverse phenotypes to genetic variations at the genome level. Furthermore, haplotype strategy integrating biological information could be used to identify sequence variants which are likely to harbor mutations affecting complex traits.

## Data Availability Statement

Publicly available datasets were analyzed in this study. This data can be found here: Dryad: doi: 10.5061/dryad.4qc06.

## Ethics Statement

The animal study was reviewed and approved by the Science Research Department of the Institute of Animal Sciences, Chinese Academy of Agricultural Sciences (CAAS) (Beijing, China). Written informed consent was obtained from the owners for the participation of their animals in this study.

## Author Contributions

LYX designed the experiments. HL analyzed the data and wrote the article. LiX, ZW, LeX, PZ, and HG collected the data. PG, YC, XG, LZ, HJG, WC, LYX, BZ, and JL discussed and improved the article. All authors read and approved the final article.

## Conflict of Interest

The authors declare that the research was conducted in the absence of any commercial or financial relationships that could be construed as a potential conflict of interest.

## Publisher’s Note

All claims expressed in this article are solely those of the authors and do not necessarily represent those of their affiliated organizations, or those of the publisher, the editors and the reviewers. Any product that may be evaluated in this article, or claim that may be made by its manufacturer, is not guaranteed or endorsed by the publisher.
