# Supplementary figures and images for: Genomic Prediction Using LD-Based Haplotypes Inferred From High-Density Chip and Imputed Sequence Variants in Chinese Simmental Beef Cattle
Source: Front Genet. 2021 Jul 29;12:665382. doi: 10.3389/fgene.2021.665382 (PMC8358323; doi:10.3389/fgene.2021.665382)

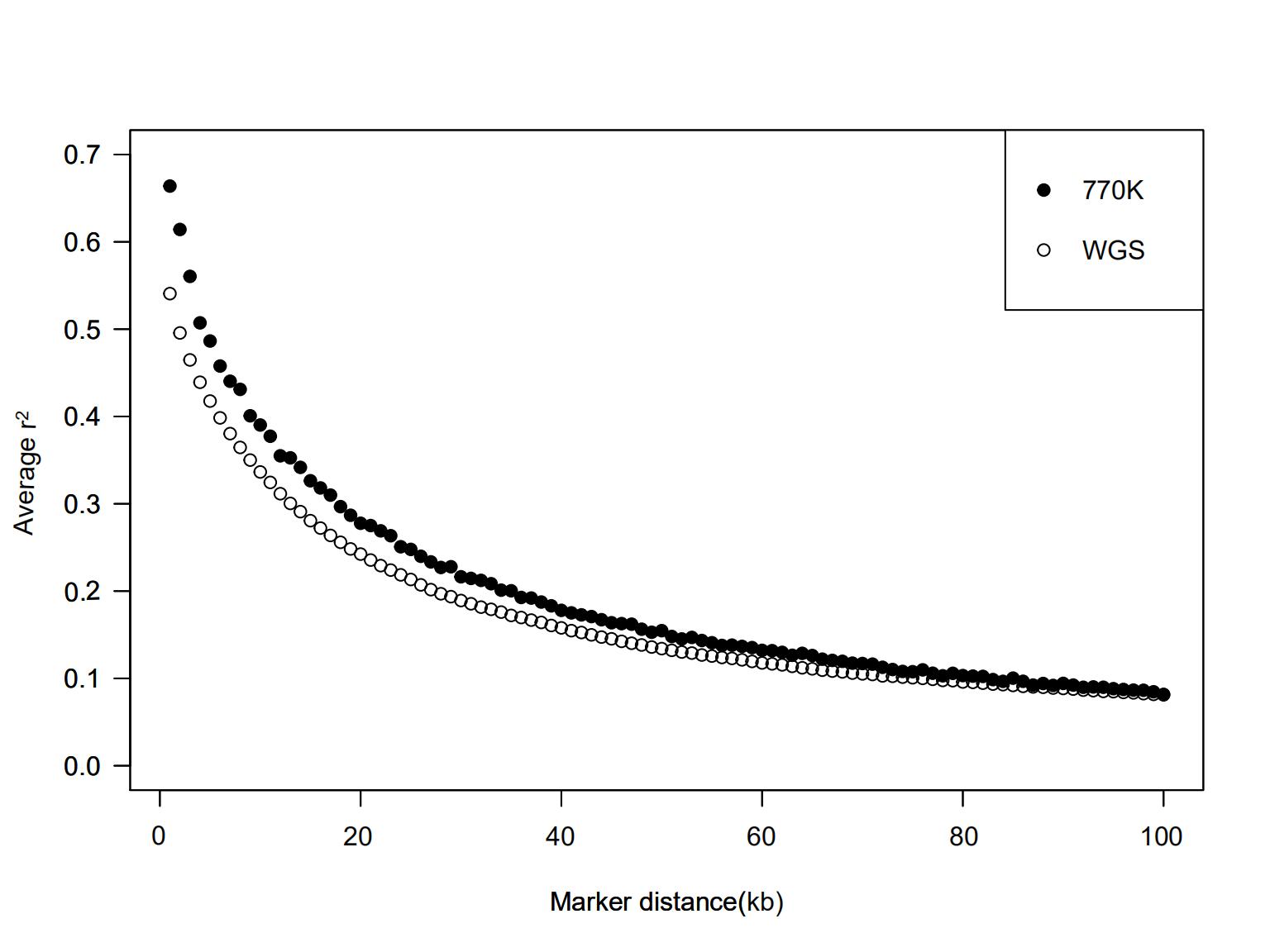


**Figure S1** **|** The LD decay of BTA1 in the 770K data and WGS data

Supplement: Supplementary file 1 [file Data_Sheet_1.docx]
