# Supplementary material for: Genomic Prediction Using LD-Based Haplotypes Inferred From High-Density Chip and Imputed Sequence Variants in Chinese Simmental Beef Cattle
Source: Front Genet. 2021 Jul 29;12:665382. doi: 10.3389/fgene.2021.665382 (PMC8358323; doi:10.3389/fgene.2021.665382)
Supplement: Supplementary file 2 [file Data_Sheet_2.docx]

**TABLE S1 |** Predictive accuracies for 3 traits in Chinese Simmental beef cattle. DP: Dressing percentage; MP: Meat percentage; RERW: Rib eye roll weight.

| **Trait** | ${\boldsymbol{r}^{\boldsymbol{2}}}^{1}$ | **GBLUP** | | **G_H_BLUP** | | **G_H_BLUP+GBLUP** | |
| --- | --- | --- | --- | --- | --- | --- | --- |
|  |  | **770K** | **WGS** | **770K** | **WGS** | **770K** | **WGS** |
| DP | - | 0.382 | 0.371 | - | - | - | - |
|  | 0.2 | 0.365 | 0.370 | 0.380 | 0.375 | 0.387 | 0.370 |
|  | 0.3 | 0.364 | 0.370 | 0.377 | 0.372 | 0.387 | 0.368 |
|  | 0.4 | 0.362 | 0.369 | 0.371 | 0.370 | 0.388 | 0.368 |
|  | 0.5 | 0.357 | 0.369 | 0.372 | 0.370 | 0.389 | 0.369 |
|  | 0.6 | 0.355 | 0.368 | 0.364 | 0.369 | 0.389 | 0.368 |
|  | 0.7 | 0.355 | 0.369 | 0.363 | 0.368 | 0.387 | 0.369 |
|  | 0.8 | 0.350 | 0.367 | 0.359 | 0.366 | 0.386 | 0.367 |
| MP | - | 0.296 | 0.286 | - | - | - | - |
|  | 0.2 | 0.278 | 0.288 | 0.290 | 0.290 | 0.301 | 0.288 |
|  | 0.3 | 0.280 | 0.290 | 0.291 | 0.289 | 0.296 | 0.288 |
|  | 0.4 | 0.277 | 0.289 | 0.292 | 0.287 | 0.295 | 0.288 |
|  | 0.5 | 0.274 | 0.288 | 0.288 | 0.285 | 0.298 | 0.282 |
|  | 0.6 | 0.272 | 0.287 | 0.282 | 0.285 | 0.298 | 0.288 |
|  | 0.7 | 0.272 | 0.287 | 0.287 | 0.287 | 0.292 | 0.288 |
|  | 0.8 | 0.260 | 0.283 | 0.278 | 0.283 | 0.295 | 0.279 |
| RERW | - | 0.406 | 0.391 | - | - | - | - |
|  | 0.2 | 0.400 | 0.383 | 0.407 | 0.397 | 0.399 | 0.396 |
|  | 0.3 | 0.398 | 0.382 | 0.406 | 0.389 | 0.398 | 0.395 |
|  | 0.4 | 0.395 | 0.380 | 0.409 | 0.389 | 0.399 | 0.395 |
|  | 0.5 | 0.397 | 0.380 | 0.409 | 0.391 | 0.399 | 0.388 |
|  | 0.6 | 0.394 | 0.380 | 0.404 | 0.389 | 0.396 | 0.394 |
|  | 0.7 | 0.393 | 0.379 | 0.402 | 0.389 | 0.395 | 0.393 |
|  | 0.8 | 0.387 | 0.377 | 0.402 | 0.389 | 0.398 | 0.396 |

^1^The seven different LD thresholds set from $r^{2}\geq0.2$ to $r^{2}\geq0.8$.

**TABLE S2 |** Regression coefficients of pre-adjusted phenotypes on GEBVs for 3 traits in Chinese Simmental beef cattle. DP: Dressing percentage; MP: Meat percentage; RERW: Rib eye roll weight.

| **Trait** | ${\boldsymbol{r}^{\boldsymbol{2}}}^{1}$ | **GBLUP** | | **G_H_BLUP** | | **G_H_BLUP+GBLUP** | |
| --- | --- | --- | --- | --- | --- | --- | --- |
|  |  | **770K** | **WGS** | **770K** | **WGS** | **770K** | **WGS** |
| DP | - | 1.043 | 1.051 | - | - | - | - |
|  | 0.2 | 1.040 | 1.051 | 1.039 | 1.053 | 1.039 | 1.024 |
|  | 0.3 | 1.041 | 1.050 | 1.044 | 1.054 | 1.042 | 1.038 |
|  | 0.4 | 1.037 | 1.051 | 1.042 | 1.055 | 1.049 | 1.038 |
|  | 0.5 | 1.036 | 1.051 | 1.044 | 1.055 | 1.049 | 1.044 |
|  | 0.6 | 1.034 | 1.052 | 1.043 | 1.055 | 1.050 | 1.026 |
|  | 0.7 | 1.032 | 1.053 | 1.042 | 1.055 | 1.048 | 1.040 |
|  | 0.8 | 1.027 | 1.053 | 1.038 | 1.057 | 1.045 | 1.039 |
| MP | - | 1.093 | 1.112 | - | - | - | - |
|  | 0.2 | 1.109 | 1.092 | 1.096 | 1.105 | 1.064 | 1.100 |
|  | 0.3 | 1.111 | 1.090 | 1.096 | 1.103 | 1.061 | 1.110 |
|  | 0.4 | 1.115 | 1.090 | 1.104 | 1.103 | 1.059 | 1.110 |
|  | 0.5 | 1.120 | 1.089 | 1.101 | 1.103 | 1.066 | 1.086 |
|  | 0.6 | 1.121 | 1.089 | 1.102 | 1.103 | 1.069 | 1.110 |
|  | 0.7 | 1.105 | 1.086 | 1.095 | 1.100 | 1.051 | 1.100 |
|  | 0.8 | 1.121 | 1.088 | 1.100 | 1.100 | 1.059 | 1.085 |
| RERW | - | 1.138 | 1.151 | - | - | - | - |
|  | 0.2 | 1.140 | 1.155 | 1.138 | 1.148 | 1.102 | 1.136 |
|  | 0.3 | 1.141 | 1.158 | 1.143 | 1.148 | 1.118 | 1.134 |
|  | 0.4 | 1.142 | 1.158 | 1.141 | 1.148 | 1.110 | 1.136 |
|  | 0.5 | 1.141 | 1.159 | 1.138 | 1.148 | 1.111 | 1.141 |
|  | 0.6 | 1.142 | 1.159 | 1.140 | 1.148 | 1.112 | 1.134 |
|  | 0.7 | 1.142 | 1.160 | 1.138 | 1.148 | 1.110 | 1.134 |
|  | 0.8 | 1.149 | 1.162 | 1.145 | 1.148 | 1.115 | 1.143 |

^1^The seven different LD thresholds set from $r^{2}\geq0.2$ to $r^{2}\geq0.8$.
